# Recording Cortical Somatosensory Responses During Cross‐Country Skiing: A Feasibility and Reproducibility EEG Study

**DOI:** 10.1111/ejn.70620

**Published:** 2026-07-09

**Authors:** Alessandra Giangrande, Alberto Botter, Giacinto Luigi Cerone, Magdalena Karczewska‐Lindinger, Stefan Lindinger, Jarmo M. Piirainen, Harri Piitulainen

**Affiliations:** ^1^ Faculty of Sport and Health Sciences University of Jyväskylä Jyväskylä Finland; ^2^ Polytechnic of Turin Turin Italy; ^3^ Laboratory of Biomechanics of Skiing Bormio Italy; ^4^ Sport Technology Unit Vuokatti, Faculty of Sport and Health Sciences University of Jyväskylä Jyväskylä Finland

**Keywords:** motor control, somatosensory processing, wireless electroencephalography

## Abstract

Human motor control emerges from the integrated activity of muscles, spinal circuits and cortical and subcortical brain structures. While simultaneous electroencephalographic and electromyographic recordings offer a powerful approach to assess the neuromuscular function across multiple levels, high‐quality cortical measurements during dynamic whole‐body movements remain technically challenging. In this study, we evaluated the feasibility and reproducibility of somatosensory evoked potentials (SEPs) recorded during V2 skate‐skiing. We also examined whether cortical responses are modulated across specific sub‐phases of the skiing cycle. Fourteen amateur skiers (13 males, 38 ± 8 years old) performed indoor treadmill skiing while receiving supramaximal electrical stimulation of the right tibial nerve at four timings of the gliding phase of the skiing cycle. The experimental protocol was repeated twice, 2 days apart, and it involved the simultaneous recording of wireless EEG, EMG and ski‐mounted forces. Our findings indicated a robust cortical response in terms of amplitude and cortical location of the peak SEPs. SEP amplitudes demonstrated a moderate to excellent between session reproducibility (ICC > 0.83, Spearman r > 0.52) with no effect of the skiing cycle sub‐phases (*p* > 0.05), indicating that electrically evoked SEPs primarily reflect low‐level cortical processing of somatosensory afference that is minimally influenced by the ongoing motor‐related cortical activity. This experimental design enables the characterization of sensorimotor integration during a whole‐body dynamic task, offering new insights into the cortical mechanisms supporting skilled locomotor performance.

## Graphical abstract

We found wireless‐EEG to be feasible to record and follow somatosensory evoked potentials during a highly dynamic naturalistic motor task, and that the cortical somatosensory processing was only minimally affected by the phase of the ongoing motor task.

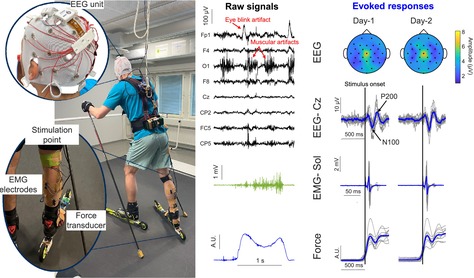

**Abbreviations**
CV: coefficient of variation
EEG: electroencephalography
EMG: electromyography
EOG: electrooculogram
GM: gastrocnemius medialis
ICC: intraclass correlation coefficient
MoBI: mobile brain/body Imaging
SEP: somatosensory evoked potential
SOL: soleus
TA: tibialis anterior

## 1 Introduction

The human motor control operates at all levels of the sensorimotor system, including the muscles, the spinal cord, the subcortical brain structures and cortex of the brain (Riemann and Lephart 2002). All levels operate jointly to produce motor actions, and different non‐invasive techniques can be used to record the related neurophysiological activity during the motor actions.

Electroencephalography (EEG) has been used to examine cortical brain activity tracking the neurophysiological modulations related to the functions of the somatosensory system in different conditions (Macerollo et al. 2018). As an example, the recording of somatosensory evoked potentials (SEPs) has been applied to monitor the cortical modulations during different motor tasks such as active vs. passive limb movements (Abbruzzese et al. 1981; Ambalavanar et al. 2025; Tinazzi et al. 1997), fine motor actions (Mongold et al. 2024), walking or gait initiation (Mouchnino et al. 2015), revealing task‐dependent gating or facilitation of somatosensory pathways.

On the other hand, the muscular level has been widely examined in various motor actions using electromyography (EMG), typically recorded from the skin surface of the given target muscle(s) (Merletti and Farina 2016). EMG is typically recorded in response to volitional activations or electrical stimuli of the peripheral motor nerves activating the target muscle, which allows the estimation of maximal motor output of the given muscle or muscle group (Bergquist et al. 2011; Doucet et al. 2012). The motor response to electrical stimulation can be quantified through the M‐wave, which represents the compound muscle action potential resulting from the stimulation of efferent α‐motoneurons innervating the muscle (Merletti and Farina 2016; Rodriguez and Nicolas 2018). M‐wave starts ~10 ms after an electrical stimulus of the peripheral nerve and its amplitude reflects the number of motor unit electrically activated (Lienhard 2015; Palmieri et al. 2004).

Therefore, simultaneous recordings of EMG and EEG allow studies of motor control at multiple levels of the neuromuscular system. Indeed, Mobile Brain/Body Imaging (MoBI) was introduced to enable the simultaneous recording of brain activity and body dynamics during natural behaviour, allowing the investigation of cognition in ecologically valid settings beyond traditional stationary paradigms (Gramann et al. 2011; Makeig et al. 2009). Early studies further demonstrated the feasibility of acquiring reliable EEG and event‐related potentials during active behaviours such as standing and walking using mobile recording systems (Gramann et al. 2010; Legrand et al. 2024; De Sanctis et al. 2012). However, this potential is still not fully exploited yet as the cortical level is the most challenging to investigate during dynamic motor actions, because cortical signals are characterized by a low signal to noise ratio and thus susceptible to motion artefacts due to several factors ranging from the quality of the electrode‐skin interface to the conditioning electronics (Giangrande, Botter, et al. 2024). In this regard, the lack of suitable instrumentation for recording of high‐quality EEG signals under unconstrained naturalistic conditions has long represented a major issue (Giangrande, Botter, et al. 2024), which partly explains why the brain basis of motor control during dynamic and sport‐related tasks remains poorly understood. Complex whole body sports, such as cross‐country skiing, requires multilevel sensorimotor integration and muscular coordination, controlled by the motor skill encoded to the brain neuronal circuits. These processes are guided by visual, vestibular and proprioceptive sensory feedback enabling continuous rapid postural adjustments as well as ongoing motor learning (Grace Gaerlan et al. 2012; Wong et al. 2012). Thus, to unravel the cortical level mechanisms, the high quality, stable and reproducible EEG signals recorded during motor activities are a prerequisite for cross‐sectional and longitudinal studies. The assessment of the neural processing in dynamic motor environments such as during skiing can provide important insights into skill acquisition, fatigue effects and the potential for performance optimization in sports.

Thanks to recent improvements in EEG technology, it has become feasible to record high‐quality EEG signals even during whole‐body dynamic movements (Jacobsen et al. 2020; Robles et al. 2021; Roeder et al. 2020). Building on these technological developments, we designed the present study to investigate the following: (i) the feasibility and reproducibility of cortical SEPs using EEG during cross‐country skiing and (ii) the modulations of the cortical responses according to V2‐skiing early loading/gliding sub‐phases. We expected SEPs to demonstrate moderate to excellent repeatability, comparable to more static experimental conditions for somatosensory processing (Piitulainen et al. 2018, 2020). Furthermore, we hypothesized some SEP‐amplitude modulation at different phases of the skiing cycle, since the ongoing cortical motor control may influence the somatosensory processing (Hu et al. 2022).

## 2 Materials and Methods

### 2.1 Participants

Fourteen amateur but experienced skiers with no history of movement disorder or neuropsychiatric disease volunteered the study (13 males, 38 ± 8 year old, weight 74 ± 8 kg, height 176 ± 5 cm, mean ± SD). The study was conducted in accordance with the Declaration of Helsinki. The University of Jyväskylä's Ethics Committee approved the experiment (approval number: 369/13.00.04.00/2020) and a thorough explanation of the study methodology was given to the participants before asking them to sign the informed consent.

### 2.2 Experimental Procedure

**FIGURE 1:**
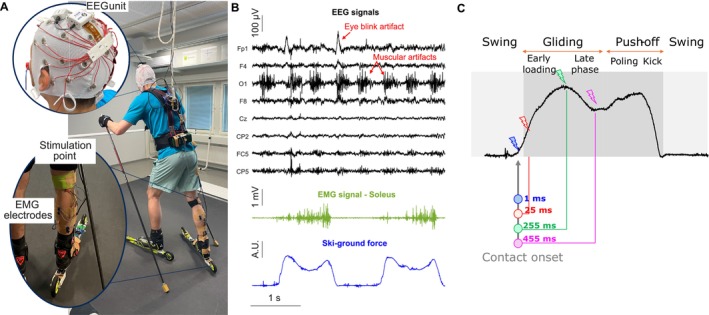
Representative signals and experimental setup. (A) Right panel: subject skiing on the treadmill equipped with EEG, EMG, ground forces and electrical stimulation instrumentation. Left panel: details of the experimental setup for the recording of brain and muscle activity. (B) Examples of recorded raw signals. From top to bottom: EEG from eight electrode sites, EMG from right soleus and ski‐ground force signals. Eye blink and muscular artefacts on EEG signals are highlighted. (C) Example of a single cycle of the ski‐ground force signal from a participant. The black trace shows the force profile over time after the contact onset (0 ms). Coloured symbols and lightning bolts indicate the timing of electric stimulations delivered at different delays (1, 25, 255, and 456 ms) relative to contact onset. Shaded areas represent the phases of the skiing cycle.

Measurements were carried out in the indoor Skiing Laboratory of the Sports Technology unit, University of Jyväskylä, Finland. The experimental procedure included supra‐maximal electrical stimulations of the right tibial nerve during cross‐country skiing on a treadmill to examine the sensorimotor processing during V2‐skiing. This skiing technique is a symmetrical skate skiing method involving a synchronous double pole push matching each leg push‐off. Supramaximal intensity was used to ensure stable maximal peripheral nerve stimulation at the different phases of the skiing contact phase. Figure 1A shows the experimental setup including the recording of EEG, EMG and ground reaction forces of the ski. The experiment was repeated twice 2 days apart to evaluate the reproducibility of the cortical SEPs. The effect of V2‐skiing gliding phase on the SEPs was investigated by delivering electrical stimuli to the tibial nerve at four different timings during the gliding phase (two stimuli during the contact at 1 and 25 ms; early gliding at 255 ms; and end gliding at 455 ms), hereinafter referred to as delays. The delays are given in ms with respect to the onset of the ski‐ground initial contact as represented in Figure 1C. The gliding phase was selected because it represents the phase in which the postural control and centre of pressure is mainly on a single ski representing the postural stability of the skiing.

#### 2.2.1 EEG Recordings

A wireless EEG amplifier (ReC Bioengineering Laboratories and LISiN, Turin, Italy, Giacinto Luigi Cerone and Gazzoni 2018) was used to record the cortical activity through 30 Ag/AgCl electrodes embedded into a cap following the international 10–20 system (EasyCap GmbH, Gliching, Germany). The electrodes sites were prepared by means of scalp abrasion (NuPrep, Weaver and Company, Aurora, United States) and gel injection (NeurGel, SPES MEDICA, Genova, Italy). Additionally, two electrooculogram (EOG) signals were acquired using surface electrodes (30 × 22 mm Ambu s.r.l., Denmark) placed following a diagonal in the eye region to recognize eye movements and blinks. Both EEG and EOG signals were recorded in monopolar derivation, using as a reference point an adhesive gelled electrode (Ø 24 mm Kendall, Covidien, Dublin, Ireland) applied on the right ear lobe after a gentle skin abrasion. EEG and EOG signals were collected with the EEG amplifier (ad resolution of 16 bits, gain of 192 V/V, sampling frequency of 2048 Hz, frequency band of 0.1 Hz—500 Hz). A wireless synchronization unit was also involved to ensure the synchronization between the recorded signals (G. L. Cerone et al. 2022).

#### 2.2.2 EMG Recordings

Bipolar surface EMG signals were collected from tibialis anterior (TA), gastrocnemius medialis (GM) and soleus (SOL) muscles of the right shank. Each muscle was investigated though a pair of Ag/AgCl electrodes (Ø 24 mm Kendall, Covidien, Dublin, Ireland) placed on the area of interest according to the electrode's placement guidelines and skin preparation (Hermens et al. 1999). Signals were collected through a wireless amplifier (Noraxon, 2400T G2, Scottsdale, United States), then they were amplified and band‐pass filtered (frequency range of 10–500 Hz) with a sampling frequency of 2000 Hz through an A/D board (CED Power 1401, CED Ltd., Cambridge, England). EMG data were recorded through Spike 6 software for further analyses (CED Ltd., Cambridge, England).

#### 2.2.3 Ski‐Ground Reaction Forces Recordings

Right ski‐ground reaction forces were recorded through a custom‐made instrumented roller ski (VTT MIKES, Technical Research Centre of Finland Ltd., Kajaani, Finland). Data was recorded in Spike 6 software with a sampling frequency of 1000 Hz through an A/D board (CED Power 1401, CED Ltd., Cambridge, England). Ski‐ground reaction forces signals were collected to identify the specific phases of the skiing cycle in order to real‐time trigger the electrical stimulation accordingly.

#### 2.2.4 Experimental Protocol

EEG, EOG and EMG electrodes were first placed and then followed by the subject preparation to the electrical stimulation. A constant‐current stimulator (DS7A, Digitimer, Hertfordshire, United Kingdom) was used to deliver electrical stimuli to the right tibial nerve. To this end, a circular cathode (Unilect short‐term ECG Electrodes, Ag/AgCl, Unomedical Ltd., Great Britain) was placed over the popliteal fossa, and an oval shaped anode (5.08 × 10.16 cm, V‐trodes neurostimulation electrodes, Mattler Electronics Corp., United States) was placed above the patella. Electrical stimuli were generated as rectangular pulses with 200 μs duration, while their amplitude was set individually according to the stimulation intensity eliciting maximal M‐waves measured from the soleus muscle during a standing rest condition at the beginning of the experiment. The supramaximal current intensity was defined as 150% of the amplitude inducing the maximal M‐wave recorded at the level of the soleus muscle. After a 5‐min warm‐up skiing, participants were asked to perform 3 min of skiing on a treadmill (Rodby, Sweden) using V2‐technique, at 2° elevation and at a velocity matching the 85% of their self‐reported maximal heart rate (Polar Vantage V2, Kempele, Finland). Participants received supramaximal electrical stimulations while skiing. Electrical stimuli were delivered every three to five (minimum of 8 s) skiing cycles with four different delays with respect to the initial onset of the gliding phase (i.e., at 1, 25, 255 and 455 ms), yielding a total of 15 stimuli for each delay. The Neurolog width/delay unit (Digitimer NeuroLog NL405, Digitimer, Hertfordshire, United Kingdom) was used to identify the rising edge of the ground force profile and to deliver the electrical stimulations at the desired timings. Specifically, an experimentally based absolute threshold of 50 N was used to define the onset of the gliding phase. With respect to the identified time instant (i.e., 0 ms), a timer provides the stimuli at the specific selected delays of 1, 25, 255 and 455 ms. The same experimental protocol was repeated twice, 24‐h apart (i.e., Day‐1 and Day‐2), to evaluate the reproducibility of the responses across sessions. In each session, stimulation protocols were administered with brief 1–2 min breaks between blocks to ensure participant comfort and maintain data quality. The order of the stimulation delay was randomized across participants and measurement sessions to avoid any systematic or time dependent confounding factors.

### 2.3 Signal Analysis

Data were processed in Matlab (Version 2024b, Mathwork Inc., Natick, MA, United States). After preprocessing, SEPs were extracted from continuous EEG signals, and M‐waves from EMG signals through averaging techniques across the electrical stimuli.

#### 2.3.1 Signal Preprocessing

EEG and EMG signals were visually inspected to identify and mark low quality signals due, for example, to poor electrode‐skin contact and/or strong movement artefacts. Signals were then filtered according to their signal bandwidths (4th order Butterworth filter, EEG: 2–95 Hz, EMG: 20–400 Hz). EEG signals were further pre‐processed by applying Independent Component Analysis to identify and discard the components associated with artefacts such eye blinks, eye movements or muscular artefact contamination from the temporal and cervical areas. Specifically, the physiological EMG contamination was identified in those components showing predominantly high‐frequency activity time‐locked to head movements during the skiing cycle, particularly over temporal and cervical regions. The above identified components were then excluded for the continuous data reconstruction. After the removal of the noisy components identified through visual inspection, the signals with poor quality were interpolated by replacing them with the average of the neighbouring electrodes (Hari and Puce 2017). Although automated approaches can improve standardization and reproducibility, a visual inspection was adopted in this preliminary investigation to allow conservative evaluation of components acquired with the newly developed wireless EEG system and to minimize the risk of inadvertent rejection of physiologically meaningful activity. EEG signals were offline referenced by applying a common average reference (i.e., subtracting at each electrode level the average of all the EEG signals). Finally, EEG, EMG and force signals were offline synchronized with kinematic data by means of a common external wireless trigger introduced in (G. L. Cerone et al. 2022). Artefact handling was intentionally performed manually, avoiding automated algorithms. Given that this is one of the first EEG studies during skiing using a novel device, a conservative approach was adopted to prevent excessive or blind removal of data.

#### 2.3.2 EEG Averaging

Continuous EEG signals were segmented into epochs from −100 to 500 ms around the stimulus onset occurring at 0 s (*n* = 15 epochs). A baseline correction was additionally performed by subtracting the average amplitude of the pre‐stimulus window signal. Epochs were averaged separately per channel, participant, delay and measurement session. The grand average SEPs were then obtained by averaging the responses across all the participants, separately for each delay and session. We characterized the peak responses at each electrode level by determining the peak amplitude of the most prominent negative and positive deflections. Because of the biphasic shape of the response, the peak to peak (maximum–minimum) amplitude was extracted to characterize the amplitude of the evoked responses.

#### 2.3.3 EEG Topography

The topographic distribution of peak amplitude was further visualized to show the spatial distribution of the evoked responses over the scalp across participants. To this end, SEPs were further low‐pass filtered (4th order Butterworth filter, cut‐off frequency 10 Hz), to avoid any possible bias of the high‐frequency residual muscular artefact contamination in the temporal and neck regions associated with typically stronger signal amplitudes (~ hundreds of μV), although the application of independent‐component analysis. This analysis was performed exclusively for illustrative purposes only and does not represent a precise source localization, as individual structural magnetic resonance imaging scans were not available.

#### 2.3.4 EMG Averaging

Continuous EMG signals were segmented into epochs from −50 to 50 ms around the stimulus onset occurring at 0 s (*n* = 15 epochs). The grand average muscular responses were then obtained by averaging the responses across all the participants, separately for each delay and session. We characterized the responses by identifying the M‐wave and the relative peak to peak (maximum‐minimum) amplitude by looking for the most prominent peak after the stimulus onset in the expected time window, i.e., 5–30 ms (Merletti and Farina 2016).

### 2.4 Statistical Analysis

Statistical analysis were performed to evaluate the day‐to‐day reproducibility, and to examine the effect of phase of skiing cycle on the cortical sensorimotor processing, i.e., SEP amplitude. Results are provided as mean ± SD The hypothesis of the normal distribution of the data was investigated by applying a Shapiro–Wilk test. Statistical analysis were performed in Matlab (Version 2024b, Mathwork Inc., Natick, MA, United States).

#### 2.4.1 Reproducibility of the Cortical and Muscle Responses

The values of SEP and M‐wave amplitudes were not normally distributed, and therefore the nonparametric Wilcoxon test was used to test differences of the means between the measurement days. Correlations of SEPs and M‐waves between the measurement days were determined with a Spearman correlation coefficient test and a two‐way mixed‐effects model intraclass correlation coefficient (ICC) was also computed between the Day‐1 and Day‐2 values to examine the reproducibility of the responses between days. ICCs were calculated using a two‐way mixed‐effects model for absolute agreement, based on single measures, providing a reproducibility estimate across sessions. The distribution of Day‐1 and Day‐2 values is represented through boxplots, correlation scatter plots and Bland Altman plots displaying the coefficient of variation (CV) between measurement days.

#### 2.4.2 Effect of Skiing Phase on the Cortical and Muscle Responses

The modulations of SEPs and M‐waves according to the skiing phases were tracked taking into account the data separately for each measurement day and evaluating cortical and muscle responses at the different stimulation delays. To this end, a Friedman test for related samples was performed to statistically test the modulations of SEPs and M‐waves amplitudes across delays. To correct for multiple comparisons when indicated (*p* < 0.05), post hoc pairwise comparisons using Wilcoxon signed‐rank tests were conducted with Bonferroni correction.

## 3 Results

Figure 1B shows continuous EEG, EMG and ski‐ground forces for two skiing cycles from a representative participant. Overall, EEG and EMG signals were of good quality with minimal motion artefacts across the participants. EEG signals were reconstructed from the raw data by discarding 3 ± 4 independent components related to artefacts due to eye movements or muscular activity. On average, among the implemented 30 electrodes, Iz, Tp9 and Tp10 electrodes suffered from motion artefact contamination in less than 9% across participants. Such noisy channels were interpolated during the preprocessing. Being the abovementioned channels located over the neck and temporal regions, they are highly dependent on the individual subject's scalp anatomy, sometimes resulting in poor contact or prone to underneath facial muscular activity related to the performance of the movements. All data segments were retained for analysis. Therefore, the recordings were overall considered successful as only three participants out of 14 were excluded from the reproducibility analysis because of technical issues encountered in the first measurement day.

### 3.1 Cortical and Muscle Responses During Skiing

**FIGURE 2:**
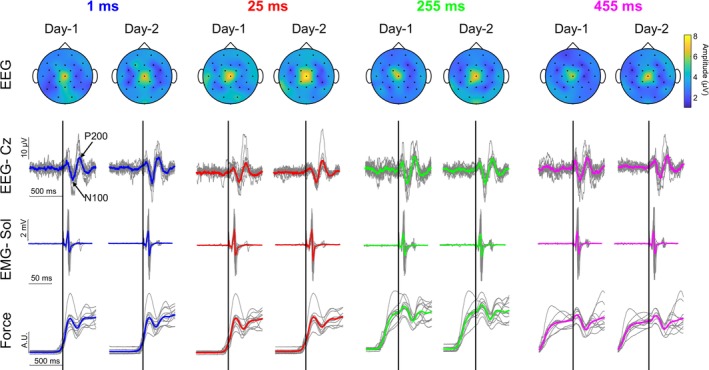
Group‐ and individual‐ level brain and muscle responses for each stimulation delay and measurement day (*n* = 15 stimuli). From top to bottom: EEG scalp topography of 2–10 Hz filtered SEPs peak‐to‐peak amplitude, SEP recorded at the Cz electrode from continuous 2–95 Hz EEG, EMG response from the SOL muscle, and ski‐ground force. The averaged EEG, EMG and ground force responses (solid lines) are superimposed on the individual responses (grey lines, *n* = 11 participants). For each response, the black vertical line indicates the stimulus onset (0 ms).

Table 1 reports the number of successfully detected SEPs and M‐waves, and the respective group‐level average amplitude for each measurement day and stimulation delay. Figure 2 shows the group‐level cortical and muscular responses during the skiing sessions for each stimulation delay (1, 25, 255 and 455 ms), and measurement day (Day‐1 and Day‐2). The topographic representation of the peak amplitude responses revealed a high reproducibility of the responses at the group level showing a robust activation of the sensorimotor cortex localized over the expected leg motor area (Cz electrode). The main N100 and P200 SEP components are prominent in the timeseries. In a few participants, an earlier, positive P50 component was also visible, but it was masked in the group average. The cortical responses were spatially highly reproducible across participants and days. M‐waves from the SOL muscle showed the typical bi‐phasic shape. However, being a supra‐maximal electrical stimulation, M‐waves were followed by a second waveform (i.e., V‐wave) in 72% of the participants' responses (across measurement days and delays) with a generally weaker amplitudes than the M‐waves.

**TABLE 1:** Summary of brain (SEPs) and muscle (M‐waves) responses to electrical stimulation during skiing. For each stimulation delay (1, 25, 255 and 455 ms) and measurement session (Day‐1 or Day‐2), the number of detectable responses, their relative peak to peak (P2P) averaged amplitudes and peak latencies (N100 and P200) are displayed (mean ± SD, across participants).

	SEPs		M‐waves	
	Day‐1	Day‐2	Day‐1	Day‐2
1 ms	11/11 sub *P2P*: 16 ± 7 μV *N100*: 148 ± 24 ms *P200*: 249 ± 12 ms	11/11 sub *P2P*: 15 ± 6 μV *N100*: 127 ± 18 ms *P200*: 242 ± 11 ms	11/11 sub 4 ± 1 mV	11/11 sub 4 ± 1 mV
25 ms	11/11 sub *P2P*: 16 ± 7 μV *N100*: 155 ± 28 ms *P200*: 252 ± 8 ms	11/11 sub *P2P*: 16 ± 9 μV *N100*: 148 ± 14 ms *P200*: 246 ± 27 ms	11/11 sub 4 ± 1 mV	11/11 sub 4 ± 1 mV
255 ms	11/11 sub *P2P*: 15 ± 6 μV *N100*: 134 ± 23 ms *P200*: 241 ± 17 ms	11/11 sub *P2P*: 16 ± 7 μV *N100*: 140 ± 19 ms *P200*: 247 ± 16 ms	11/11 sub 4 ± 1 mV	11/11 sub 4 ± 2 mV
455 ms	11/11 sub *P2P*: 14 ± 6 μV *N100*: 132 ± 17 ms *P200*: 238 ± 29 ms	11/11 sub *P2P*: 16 ± 7 μV *N100*: 142 ± 12 ms *P200*: 246 ± 23 ms	11/11 sub 4 ± 1 mV	11/11 sub 4 ± 2 mV

### 3.2 Reproducibility of Cortical and Muscle Responses

**FIGURE 3:**
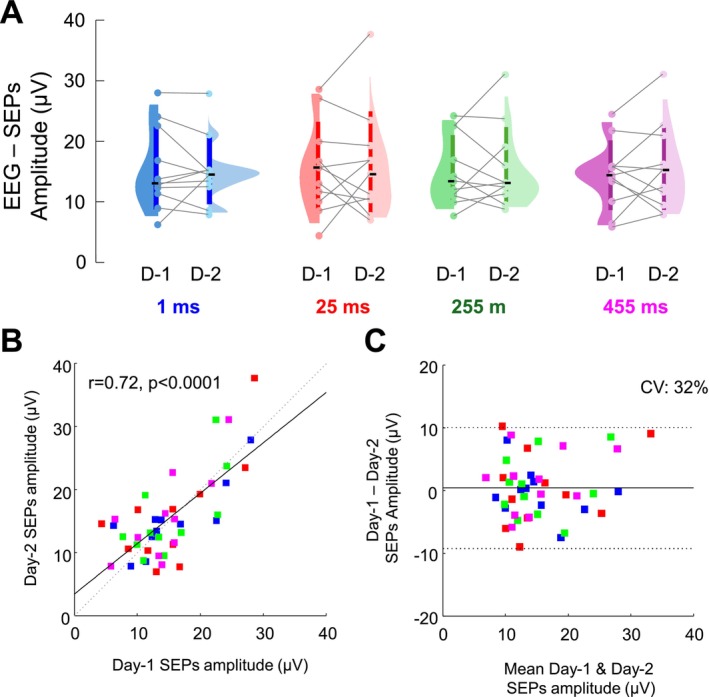
Inter‐session reproducibility of SEPs peak to peak amplitude. (A) SEPs amplitudes at group (boxplots) and individual (grey lines) level for Days 1 and 2 at each tested delay (1 ms in blue, 25 ms in red, 255 ms in green and 455 ms in purple). The shaded areas correspond to the violin plots of the probability distribution of the Cz peak to peak SEPs amplitude. (B) Correlation scatter plot of SEPs amplitude at Day‐1 and Day‐2 with data pooled from electrical stimulation delays. (C) Bland Altman plot of agreement between measurement days for SEPs amplitude. The solid black line indicates the mean difference between measurement days and dashed lines correspond to 95% confidence interval. The coefficient of variation (CV) is displayed.

All results on inter‐session reproducibility are summarized in Table 2. Figure 3 illustrates the reproducibility results of individual SEPs across measurement days for each tested stimulation delay. The distributions of SEPs amplitudes shown in Figure 3A were similar between measurement days for each tested delay as indicated by the overlapping interquartile ranges. Indeed, we observed no statistically significant differences in median values (*p* > 0.5 for all stimulation delays). The scatter plot of Figure 3B shows a linear trend, with most data points clustering around the best‐fit line. A significant positive correlation was observed (r = 0.72, *p* < 0.0001), indicating a strong association of SEP amplitude between the measurement days. Likewise, the Bland–Altman plot of Figure 3C revealed a moderate agreement between the two measurement days with moderate systematic errors, showing a variation rate of 32% across measurement days.

**TABLE 2:** Summary of the inter‐session reproducibility results of brain (SEPs) and muscle (M‐waves) responses to electrical stimulation during skiing.

	SEPs			M‐waves		
	ICC value	95% CI	Spearman r	ICC	95% CI	Spearman r
1 ms	0.89***	0.62–0.97	0.78**	0.89***	0.62–0.97	0.86***
25 ms	0.84***	0.38–0.96	0.52*	0.88***	0.58–0.96	0.86***
255 ms	0.83***	0.32–0.95	0.61*	0.80***	0.26–0.95	0.78**
455 ms	0.83***	0.40–0.96	0.71**	0.84***	0.42–0.96	0.83**

* *p* < 0.05.

** *p* < 0.01.

*** *p* < 0.003.

**FIGURE 4:**
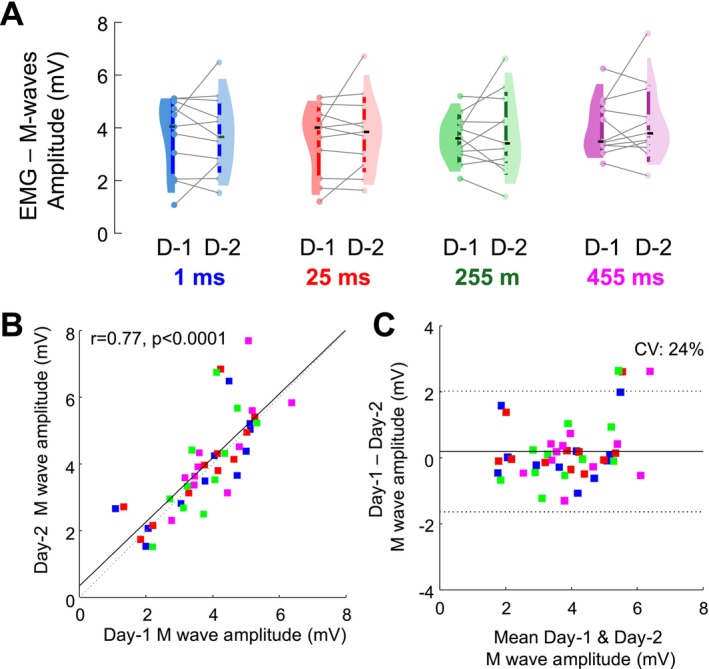
Inter‐session reproducibility of muscular responses. (A) M‐waves amplitude at group (boxplots) and individual (grey lines) level for Days 1 and 2 for each tested delay (1 ms in blue, 25 ms in red, 255 ms in green and 455 ms in purple). The shaded areas correspond to the violin plots of the probability distribution of the M‐ waves amplitude. (B) Correlation scatter plot of M‐waves amplitudes at Day‐1 and Day‐2 with data pooled from electrical stimulation delays. (C) Bland Altman plots of agreement between the measurement days for M‐waves amplitude. The solid black lines indicate the mean difference between measurement days and dashed lines correspond to 95% confidence interval. The coefficient of variation (CV) is displayed.

Similar reproducibility was observed for the M‐wave amplitude (Figure 4A–C). Figure 4A shows the boxplot representation of the M‐wave amplitudes that were well overlapping between measurement days for all stimulation delays with no significant differences (*p* > 0.8 for all stimulation delays). The scatter plot of Figure 4B revealed a clear linear pattern, with the majority of data points closely aligned with the trend line, and a strong positive correlation between the measurement days (r = 0.77, *p* < 0.0001). This robust association was reinforced with minimal discrepancy and low variation (24%) in day‐to‐day the M‐wave amplitude as depicted in Figure 4C.

### 3.3 Effect of Skiing Phase on the Cortical and Muscle Responses

**FIGURE 5:**
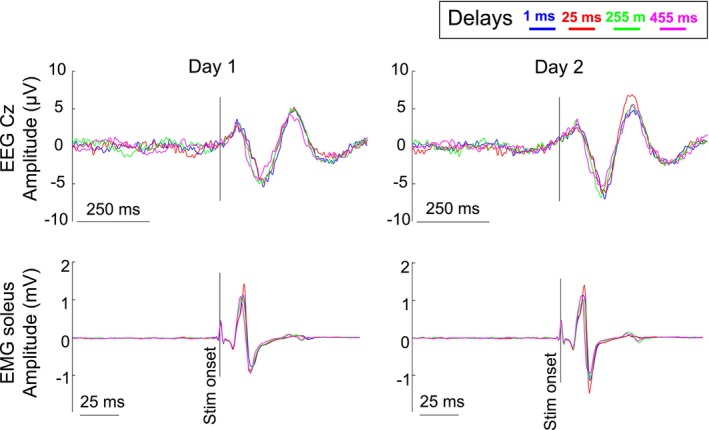
Group‐level SEP for Cz electrode (top panels) and M‐wave of SOL muscle (lower panels) responses for each stimulation delay (superimposed) and measurement day. Filter bandwidths EEG: 2–95 Hz, EMG: 20–400 Hz.

Figure 5 shows group level SEPs and M‐waves for each four‐stimulation delay on both measurement days. The SEP and M‐wave shapes remained similar across stimulation delays in both measurement days. Table 3 summarizes the Friedman test results, which did not reveal statistically significant differences in SEP amplitude between the delays (i.e., within the gliding/push‐off sub‐phases) in either measurement day.

**TABLE 3:** Summary of the Friedman statistical test on brain (SEPs) and muscle (M‐waves) amplitudes across stimulation delays, separately for measurement day.

	SEPs	M‐waves
Day‐1	χ^2^(3) = 0.05, *p* = 0.99	χ^2^(3) = 2.24, *p* = 0.53
Day‐2	χ^2^(3) = 0.27, *p* = 0.97	χ^2^(3) = 10.02, p = 0.02

Similarly, M‐waves amplitude did not show statistically significant difference between the delays on the first measurement day. On the second day, Friedman test indicated a significant difference in M‐wave amplitude between 455‐ms delay and the other tested delays (*p* = 0.02), but it was no longer significant after Bonferroni correction (all corrected *p*‐values > 0.05).

## 4 Discussion

This is the first study to demonstrate the feasibility of wireless EEG recordings during naturalistic highly dynamic and technically demanding conditions of cross‐country skiing. We showed that SEPs evoked during skiing are highly reproducible between measurement days at the group level. Thus, wireless EEG can provide reliable neurophysiological data even during complex, real‐world motor tasks. We further demonstrated that it is feasible to deliver reproducible electrical peripheral (tibial) nerve stimulations, and synchronously record the brain and muscular responses, and ground‐reaction forces during cross‐country skiing. Therefore, wireless EEG can be effectively used to quantify the cortical processing of somatosensory afferent input during dynamic tasks and longitudinal experimental designs, to investigate the neuromuscular system at multiple levels (i.e., cortical, muscular, etc.) even in sports or other highly demanding contexts. Finally, it was surprising that the SEP amplitude did not modulate during sub‐phases of the gliding/loading of the V2‐skiing. These observations may indicate that electrically elicited SEPs largely reflect low‐level cortical processing of somatosensory afference, with limited modulation by ongoing cortical motor control.

### 4.1 Feasibility and Reproducibility of Cortical Responses

Cortical responses elicited during skiing were expected in terms of SEPs amplitude, latency and cortical location of the peak response, i.e., on the Cz electrode above the leg area of the primary sensorimotor cortex. Indeed, SEP is the cortical response to electrically evoked somatosensory afferents in the tibial nerve that typically peaks in the primary somatosensory cortex following somatotopic organization (Kaas 2005). Since we used supramaximal stimulation intensity, activating the plantar flexor muscles, the SEP was likely affected by secondary delayed (with ~30 ms) volley of somatosensory afference to the cortex due to muscle contraction/twitch. Nevertheless, the main SEP peak likely reflects primarily the initial electrically evoked somatosensory afferent volley, or potentially sum effect of the both volleys, which is good to be aware when planning experimental designs using dynamic EEG recordings.

Our findings indicated a robust cortical response in terms of amplitude and cortical location of the peak SEPs in both measurement days. In addition, SEPs were stable from day to day, showing good to excellent reproducibility. This was a somewhat striking result, as there was potentially some degree of motor learning between the days, even though all participants were allowed to perform a familiarization session prior to the actual first EEG session. Thus, some motor learning might have happened, especially among the less experienced roller skiers (2 out of 11) or treadmill skiers (8 out of 11) in our sample. This result suggests that the SEPs are relatively stable even to motor learning, but this claim requires further intervention studies to be confirmed. However, it is likely that the skiers were able to perform better on the second day, which may increase the variability of the SEP responses, but we did not observe clear changes or trends to support this hypothesis.

Electrical stimulation of the peripheral somatosensory nerve in static or quasi‐static conditions has been shown to be reproducible, but typically around 100 stimuli or more are recommended to be elicited to secure the signal‐to‐noise ratio for SEPs (Cruccu et al. 2008). Thus, it was surprising that we obtained highly reproducible strong SEPs even with a low number of stimuli, only 15 stimuli per delay, during a highly dynamic full‐body motor task. This demonstrates that SEPs to supramaximal electrical stimulation activate the primary somatosensory cortex quite strongly, enabling the reproducible SEP extraction in carefully conducted dynamic experimental conditions. It is noteworthy that typically SEPs are evoked with much lower electrical stimulation intensities, even below the motor threshold, which might partly explain our robust results with only ~15 stimuli.

### 4.2 Feasibility and Reproducibility of Muscle Responses

As the cortical responses, the muscular responses did not show significant modulation with the skiing phase. As expected, the M‐wave muscle responses were indeed highly reproducible between the measurement days in terms of the response amplitude, verifying that the supramaximal nerve stimulation was successful. The stability of the M‐wave responses was also in line with previous studies on direct peripheral nerve electrical stimulation, revealing their extreme reproducibility under controlled conditions (Calder et al. 2005). It is worth mentioning that the M‐wave response could vary due to three main factors. From a physiological standpoint, the level of activation of the target muscle may vary throughout the gliding phase. Consequently, an identical electrical stimulus might activate a different number of muscle fibres depending on whether some are in their refractory period. This phenomenon could, in principle, be evaluated by examining the amplitude of the voluntary EMG activity immediately preceding the stimulus. From a methodological perspective, two additional factors could be considered. First, variations in the geometric relationship between the nerve and the stimulation electrode during the gliding phase might lead to differential axonal recruitment by the same stimulus. Nonetheless, this explanation appears unlikely, as the supramaximal stimulation employed should ensure activation of all axons irrespective of minor positional shifts of the electrode relative to the nerve. The large surface area of the stimulating electrode further supports this assumption. Second, changes in ankle and knee joint angles throughout the gliding phase may influence the length and pennation angle of the target muscles, thereby affecting the M‐wave amplitude. This effect has been previously documented for the tibialis anterior (Vieira et al. 2017) under extreme conditions, such as full ankle flexion and full extension. In the present context, however, the range of motion is considerably smaller; thus, although this potential influence cannot be entirely excluded, its impact is likely minimal given the limited angular displacement involved.

The recording of high‐quality EEG during dynamic conditions alone is a major challenge, but there were also more peripheral challenges in our experimental setup. One of the main challenges was the (re‐)placement of the stimulation electrode above the tibial nerve, and its stabilization during the skiing, since the knee joint is in constant movement during the skiing cycle (Cattagni et al. 2017). Therefore, we paid special attention to ensure a ‘constant’ pressure of the stimulation electrode on the skin by taping a pressure pad on top of the stimulating electrode; however, the effects of anatomical differences cannot be excluded. This includes, for example, the depth of the popliteal fossa and its variation at different joint angles. For this reason, we used strong supramaximal stimulation intensity to mitigate these issues, which appeared to work out well. As a result, the particular attention paid on the experimental setup to ensure enough pressure on the stimulation point was further demonstrated by the highly reproducible M‐wave muscle responses, which are highly dependent on the stability of the stimulation condition.

### 4.3 Effect of Skiing Phase on the Cortical Responses

No effect of the skiing phase on cortical response was found in our results. We hypothesized modulation of SEP‐amplitude at different phases of the early skiing cycle, since it has been shown earlier that ongoing cortical motor control may, and likely does, influence the somatosensory processing (Hu et al. 2022). However, this was not reflected in our SEPs evoked with supramaximal intensity, which may partly explain the lack of modulation in ‘all out’ SEPs. Indeed, the relatively high electrical stimulation intensity used (150% of maximal M‐wave), may have produced a ceiling effect in the activation of somatosensory afferents. Under such conditions, it is likely that most, if not all, afferent fibres were already recruited by the stimulation, resulting in a strong somatosensory input to the cortex. Consequently, potential modulation of cortical responses (i.e., SEPs) across different skiing phases may have been masked by this near‐maximal afferent input. Nevertheless, changes in cortical state during ongoing motor activity could still theoretically produce inhibitory or facilitatory effects on SEP amplitudes, for example, through modulation of cortical excitability or recruitment of different neuronal populations. Therefore, the present findings are noteworthy, since one would intuitively expect the ongoing motor action and its cortical planning/control to influence the cortical processing of the somatosensory afference, even though it is artificial and thus unrelated to the specific ongoing task. Somatosensory gating refers to the ability of the central nervous system to regulate (either suppress or enhance) sensory input, depending on the context, but particularly during movements for the somatosensory domain. This function allows the brain to prevent overload and reduce irrelevant inputs by adjusting the cortical processing according to behavioural demands (Deiber et al. 2012; Lattari et al. 2010; Proske and Gandevia 2012; Purves et al. 2018). The peripheral nerve electrical stimulation can be seen as a surprising perturbation for the sensorimotor system that must be actively attenuated to maintain smooth ongoing motor action. Our results suggest that the neurons in the primary somatosensory cortex are activated in a designated fixed manner to the electrical stimulation despite the state of the sensorimotor cortices. This may happen only if the afferent information is not functionally relevant, like in the case of electrical nerve stimulation, but is rather perceived as a distraction by the brain. However, when the afferent somatosensory afference is useful, like in the case of more naturalistic stimulation of the somatosensory receptors, the cortical response related to its processing may vary. This hypothesis has been verified in the proprioceptive domain (Giangrande, Cerone, et al. 2024; Giangrande, Mujunen, et al. 2024; Huttunen and Lauronen 2012), as we found that the cortical responses were modulated when the participant was actively performing a motor task that involved naturalistic proprioceptive stimulation (evoked movements) compared with stimulation on passive rest condition. At present, however, the influence of electrical stimulus intensity on cortical modulation under naturalistic movement conditions remains largely unexplored. Therefore, further studies with larger number and types of stimuli and motor tasks are needed to clarify how the brain utilizes and inhibits the somatosensory afference during ongoing motor actions and motor learning over longer periods of, for example, skiing (skill) training. In addition, it would be important to examine and confirm whether the cortical SEP amplitude is modulated from passive sitting/lying position to semi‐passive standing condition and to dynamic skiing conditions. Unfortunately, we did not record enough supramaximal stimuli in other than the skiing condition to answer this question with the current data. Therefore, results should be cautiously interpreted as a study on a larger sample size with a bigger amount of provided electrical stimuli might be needed to deepen the response modulations.

### 4.4 Limitations and Future Improvements

In the current study, we focused on the reproducibility of the cortical responses to supra‐maximal electrical stimulation of the tibial nerve of our participants while skiing. However, there are some factors that were not taken into consideration. Firstly, it is important to underline that EEG responses might be dependent on the individual task‐related skills of the tested participants. As an example, weaker SEPs are expected for skilled athletes since it is reasonable to hypothesize that such population activates a smaller number of neurons to initiate and control the movement with respect to amateur or non‐skiers. Secondly, further studies are also needed to analyse more thoroughly the cortical somatosensory processing and its integration with the ongoing descending motor output. Indeed, although the analysis of EMG responses was out of the scope of the present proof‐of‐concept study, combined investigations of the brain and muscle activities might shed light on the role of spinal and supraspinal motor control during cross‐country skiing, and on the related sensorimotor gating phenomena. Thirdly, despite the encouraging results in terms of the reproducibility of cortical and muscular responses, it is worth underlining the extreme importance of the methodological rigour in both the cross‐sectional and longitudinal studies, especially when dealing with challenging experimental setups such as the one hereby proposed. Among all the factors, the stability of the stimulation electrode placement is thought to significantly affect the reproducibility results and it is not possible to exclude it as a possible confounding factor also in the current study. Indeed, in our experimental design, it was not possible to ensure a stable contact between the stimulation electrode and the popliteal fossa over time as it was likely to be lost or varied in any case while skiing. Therefore, innovative approaches should be better investigated to mitigate this confounding factor in future studies such as the implementation of a spring‐like behaviour electrode compensating for the electrode's detachments during movements. Finally, the present study included a relatively small sample size, predominantly composed of male participants. Consequently, future research should involve a larger and more gender‐balanced cohort to address the limitations highlighted above and to enable more robust and generalizable conclusions.

## 5 Conclusion

This is the first study investigating SEPs during dynamic sport applications as cross‐country skiing. Despite the existing challenges of motion artefacts, multi‐signal synchronization and environmental factors, we successfully implemented a newly developed light‐weight wireless EEG in such settings, marking a significant step forward in the application of mobile neuroimaging in extreme sports. The results were particularly promising. Indeed, the extracted physiological cortical responses to the electrical stimulation revealed excellent reproducibility also during such a dynamic task as cross‐country skiing, even with a relatively low number of stimuli given. Furthermore, our results further support the hypothesis that SEPs reflect low level cortical processing of the somatosensory afference that is negligibly modulated by the ongoing cortical motor control. This finding provides the foundation for the use of these measures as neurophysiological markers to investigate adaptations in cortical processing of proprioception also in extremely dynamic scenarios.

## Author Contributions

**Alessandra Giangrande:** formal analysis, visualization, writing – original draft, writing – review and editing, investigation, data curation. **Alberto Botter:** funding acquisition, writing – review and editing, supervision, formal analysis. **Giacinto Luigi Cerone:** writing – review and editing, supervision, formal analysis. **Magdalena Karczewska‐Lindinger:** conceptualization, investigation, writing – review and editing. **Stefan Lindinger:** conceptualization, investigation, writing – review and editing. **Jarmo M. Piirainen:** conceptualization, data curation, investigation, formal analysis, funding acquisition, writing – review and editing, project administration. **Harri Piitulainen:** conceptualization, supervision, funding acquisition, writing – review and editing, writing – original draft, data curation, formal analysis.

## Funding

This work was supported by Academy of Finland, 361732 and Kainuun Rahasto, 20231386.

## Conflicts of Interest

The authors declare no conflicts of interest.

## Data Availability

The data is not publicly available due to privacy restrictions following the General Data Protection Regulation on ethical permission. However, pseudonymized data are available upon request from the corresponding author within the European Union.
